# Regulation of HTLV-1 Gag budding by Vps4A, Vps4B, and AIP1/Alix

**DOI:** 10.1186/1743-422X-4-66

**Published:** 2007-07-02

**Authors:** Shuzo Urata, Hideyoshi Yokosawa, Jiro Yasuda

**Affiliations:** 1 First Department of Forensic Science, National Research Institute of Police Science, Kashiwa 277-0882, Japan; 2 CREST, Japan Science and Technology Agency, Saitama 332-0012, Japan; 3 Department of Biochemistry, Graduate School of Pharmaceutical Sciences, Hokkaido University, Sapporo 060-0812, Japan

## Abstract

**Background:**

HTLV-1 Gag protein is a matrix protein that contains the PTAP and PPPY sequences as L-domain motifs and which can be released from mammalian cells in the form of virus-like particles (VLPs). The cellular factors Tsg101 and Nedd4.1 interact with PTAP and PPPY, respectively, within the HTLV-1 Gag polyprotein. Tsg101 forms a complex with Vps28 and Vps37 (ESCRT-I complex) and plays an important role in the class E Vps pathway, which mediates protein sorting and invagination of vesicles into multivesicular bodies. Nedd4.1 is an E3 ubiquitin ligase that binds to the PPPY motif through its WW motif, but its function is still unknown. In the present study, to investigate the mechanism of HTLV-1 budding in detail, we analyzed HTLV-1 budding using dominant negative (DN) forms of the class E proteins.

**Results:**

Here, we report that DN forms of Vps4A, Vps4B, and AIP1 inhibit HTLV-1 budding.

**Conclusion:**

These findings suggest that HTLV-1 budding utilizes the MVB pathway and that these class E proteins may be targets for prevention of mother-to-infant vertical transmission of the virus.

## Background

The Gag polyprotein of HTLV-1 is the only viral protein that is both necessary for and sufficient to drive the release of virus particles through a budding process [1-7]. During or after the process of particle release, the action of the retroviral protease cleaves Gag to produce mature matrix (MA), capsid (CA), and nucleocapsid (NC) proteins. Three functional domains that are critical for the assembly and budding processes have been identified in the Gag protein. The membrane-binding domain (M-domain) is required for myristoylation of the Gag N-terminal region and subsequent targeting of the protein to the plasma membrane. The interaction domain (I-domain) appears to be a major region involved in Gag multimerization. The late assembly domain (L-domain) plays a critical role in pinching off of virus particles from the plasma membrane of infected cells. It has also been reported that inactivation of the viral protease has no effect on the production of HTLV-1 particles, similar to our previous observations in Mason-Pfizer monkey virus (M-PMV) [7,8].

Three L-domain consensus sequences, PPXY, PT/SAP, and YPXL, have been identified within the matrix proteins of many enveloped RNA viruses, including retro-, rhabdo-, filo-, and arenaviruses [1,2,4,5,9-21]. The majority of retroviruses possess PPXY and/or PT/SAP motifs as an L-domain, one exception being equine infectious anemia virus (EIAV), which possesses a YPXL motif. Most of the host factors that interact with the L domain are involved in the class E vacuolar protein-sorting pathway, suggesting that budding into the lumen of multivesicular bodies (MVBs) in late endosomes and viral budding at the plasma membrane are topologically identical and share a common mechanism. Three ESCRT complexes, ESCRT-I, -II, and – III, play critical roles in the MVB sorting pathway, acting in a sequential manner. In the final step of protein sorting, AAA-type ATPase Vps4A/B interacts with ESCRT-III to catalyze disassembly of the ESCRT machinery to recycle its components.

The PTAP motif was first identified in human immunodeficiency virus (HIV) p6Gag and has been reported to interact with Tsg101, which is a ubiquitin-conjugating E2 variant and participates in vacuolar protein-sorting (Vps) machinery. The interaction between p6Gag and Tsg101 is required for HIV-1 budding, and Tsg101 appears to facilitate this budding by linking the p6 late domain to the Vps pathway [22,23].

The PPXY motif has been shown to be the core sequence involved in binding to the WW domain, a sequence of 38 to 40 amino acids containing two widely spaced tryptophan residues, which are involved in protein-protein interaction. In fact, it has been shown that the viral PPXY sequences interact with the WW domains of the cellular Nedd4-like ubiquitin ligases, such as Nedd4 and BUL1 [4,24-26].

The YPXL motif in EIAV p9 and a related sequence YPLASL in HIV-1 p6 have been shown to interact with AIP1/Alix, which has been reported to be linked to ESCRT-I and -III [23,27-29].

It has been reported that expression of dominant negative (DN) forms and small interfering RNA (siRNA) specific for Tsg101 and AIP1/Alix inhibit L-domain-mediated VLPs or virus release [3,12,13,18,22,30]. In addition, in many cases, DN forms of Vps4A and Vps4B lacking the ability to bind or hydrolyze ATP were shown to inhibit the budding of VLPs or virions [9,10,12,22,23,31,32].

In this study, to investigate the mechanism of HTLV-1 budding in detail, we analyzed HTLV-1 budding using DN forms of the class E proteins. Our results showed that the DN forms of Vps4A, Vps4B, and AIP1 markedly suppressed VLP production, suggesting that HTLV-1 budding utilizes the MVB pathway and that these class E proteins may be the targets for prevention of mother-to-infant vertical transmission.

## Results and Discussion

### HTLV-1 Gag budding utilizes Vps4A and Vps4B

**Figure 1:**
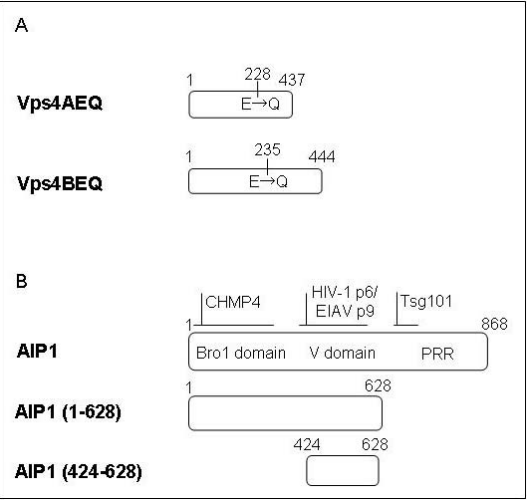
**DN forms of class E proteins used in this experiment**. A. DN forms of Vps4A and Vps4B. Both Vps4AEQ and Vps4BEQ have point mutations that render them defective in ATP hydrolysis. B. DN forms of AIP1/Alix. AIP1/Alix is composed of three major domains: Bro1 domain, V domain, and PRR (proline-rich region) [27]. The role of AIP1/Alix in endosomal sorting and virus budding requires binding the ESCRT-III component CHMP4 at the broad Bro1 domain and the ESCRT-I component Tsg101 at the PRR. HIV-1 p6 and EIAV p9 bind to the V domain.

**Figure 2:**
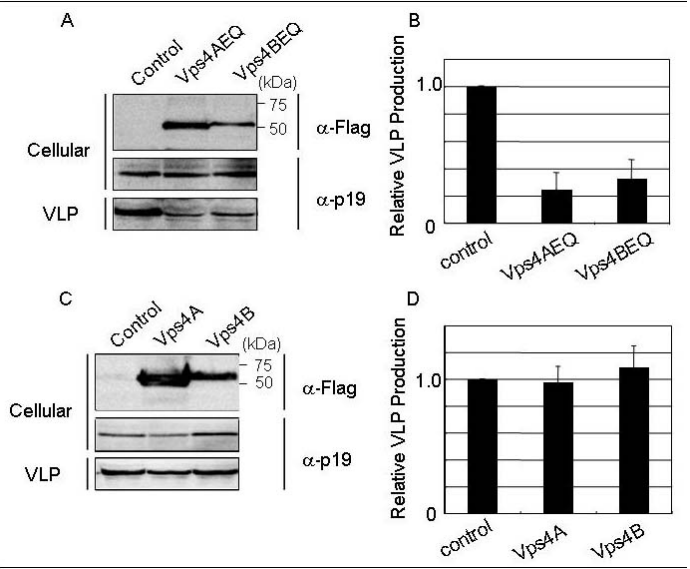
**The involvement of Vps4A and Vps4B in HTLV-1 Gag budding**. A. 293T cells were cotransfected with pK30-Gag and the expression plasmid for Vps4AEQ or Vps4BEQ, or the empty vector as a control. Extracellular VLPs were pelleted from the culture fluids. VLP-associated or cell-associated Gag was detected by western blotting (WB) using anti-HTLV-1 p19 monoclonal antibody. C. 293T cells were cotransfected with pK30-Gag and the expression vector for wild-type Vps4A or Vps4B, or the empty vector as a control. The proteins were detected as described in A. B and D. Intensities of the bands corresponding to cell- and VLP-associated Gag in A and C were quantified using the LAS3000 imaging system (Fuji film). The efficiency of Gag-induced VLP budding in cells cotransfected with pK30-Gag and control vector (VLP/Cellular) was set to 1.0. The data represent averages and standard deviations (SD) of 3 independent experiments.

Vps4A and Vps4B are ATPases, each of which is the final effector in the MVB sorting pathway in cells. Recent studies using DN of Vps4A have shown that activity of this enzyme is required for efficient budding of HIV-1, murine leukemia virus, equine infectious anemia virus, Mason-Pfizer monkey virus, simian virus 5, vesicular stomatitis virus (VSV), human hepatitis B virus, Ebola virus, and Lassa virus [10,12,22,31-35]. In contrast to Vps4A, the contribution of Vps4B in virus budding has not been demonstrated, although we previously showed that Lassa virus budding utilizes Vps4B [12]. To examine the involvement of Vps4A and Vps4B in the egress of HTLV-1 Gag-induced VLP, we analyzed the effects of overexpression of DN mutants of Vps4A and Vps4B, termed Vps4AEQ and Vps4BEQ, respectively (Fig. 1A) [12]. Both DN mutants were expressed as proteins containing a Flag tag at their N-termini. As shown in Fig. 2A and 2B, HTLV-1 Gag-induced VLP production was significantly reduced by the overexpression of Vps4AEQ or Vps4BEQ. Relative levels of production of VLPs from cells expressing Vps4AEQ and Vps4BEQ were 25% and 33%, respectively (Fig. 2B). To further examine the effects of overexpression of wild-type Vps4A and Vps4B, we also cotransfected pVps4A and pVps4B with pK30-Gag into 293T cells. As shown in Fig. 2C and Fig. 2D, overexpression of Vps4A and Vps4B did not promote VLP production. These results indicate that endogenous Vps4A and Vps4B are sufficient for producing VLPs, but the enzymatic activities of Vps4A and Vps4B are clearly required for efficient budding of HTLV-1.

### DN form of AIP1/Alix suppresses the egress of the HTLV-1 VLP production

**Figure 3:**
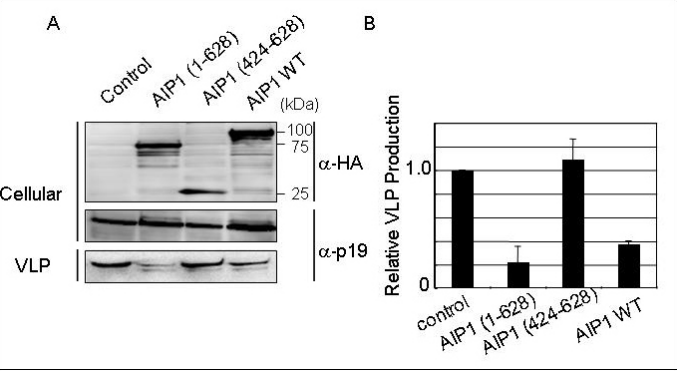
**Effects of AIP1/Alix DN mutants on HTLV-1 VLP production**. A. 293T cells were cotransfected with pK30-Gag and the expression plasmid for AIP1 (1–628), AIP1 (424–628), or AIP1 WT, or the empty vector as a control. Extracellular VLPs were pelleted from the culture fluids. VLP- or cell-associated Gag was detected by WB using anti-p19 monoclonal antibody. B. Intensities of the bands corresponding to cell- and VLP-associated Gag in A were quantified using the LAS3000 imaging system (Fuji Film). The efficiency of Gag-induced VLP budding in cells cotransfected with pK30-Gag and the control vector (VLP/Cellular) was set to 1.0. The data represent averages and standard deviations (SD) of 3 independent experiments.

To examine the involvement of AIP1/Alix in HTLV-1 budding, we overexpressed mutant forms of AIP1/Alix with pK30-Gag (Fig. 1B). As shown in Fig. 3A and 3B, the AIP1/Alix mutant AIP1 (1–628) significantly inhibited the production of HTLV-1 Gag-induced VLP. On the other hand, another mutant of AIP1/Alix, AIP1 (424–628), had no effect. Overexpression of WT AIP1/Alix suppressed the Gag-induced VLP production. Although we could not detect the interaction between HTLV-1 Gag and AIP1 (data not shown), AIP1 may regulate HTLV-1 budding indirectly. Similar results were obtained in a previous study examining the involvement of Tsg101 in HIV-1 budding [36]. Overexpression of the wild-type and C-terminal deletion mutant of Tsg101 inhibited HIV-1 production. The intracellular levels of Tsg101 appear to be strictly regulated for its physiological function. AIP1/Alix may be subject to similar regulation in cells.

The mechanism responsible for HTLV-1 budding has not been addressed in detail. Previous studies showed that HTLV-1 Gag protein plays a key role in viral budding as in other retroviruses, and that Tsg101 and Nedd4.1 recognize PTAP and PPPY within the HTLV-1 Gag polyprotein, respectively. Although it is well known that Tsg101 and Nedd4.1 play important roles in HTLV-1 budding, further mechanisms have not been characterized. In this study, to investigate the mechanism of HTLV-1 budding in detail, we analyzed HTLV-1 budding using DN forms of the class E proteins Vps4A, Vps4B, and AIP1 (Fig. 1). The results indicated that the catalytic activities of Vps4A and Vps4B are required for budding of HTLV-1 VLPs, suggesting that HTLV-1 budding mimics the MVB pathway, similar to observations in other envelope viruses. The DN form of AIP1 expressing only the N-terminal region from residues 1–628 also suppressed the budding of HTLV-1 VLPs. The Bro1 domain of AIP1, which is present in the N-terminal region, has been reported to interact with CHMP4 [23,33]. PRR binds to Tsg101. The V domain can bind to HIV-1 p6 and EIAV p9, and overexpression of a mutant containing only the V domain suppresses HIV-1 and EIAV particle release [28,29]. Our results shown in Fig. 3 can be explained by binding of AIP1 (1–628) to CHMP4, thus disturbing the downstream parts of the MVB pathway. On the other hand, AIP1 (424–628) had no effect on HTLV-1 budding. AIP1 (424–628) appears to be sufficient for the function of AIP1 in HTLV-1 budding, suggesting that HIV-1 and HTLV-1 utilize AIP1 in different ways [28,29].

Taken together, these results strongly suggest that HTLV-1 budding utilizes the MVB pathway and that these class E proteins may be useful as targets for prevention of mother-to-infant vertical transmission.

## Conclusion

In the present study, we showed that the enzymatic activities of Vps4A and Vps4B are required for efficient budding of HTLV-1 and that endogenous Vps4A and Vps4B are sufficient for VLP production. In addition, it was shown that AIP1 (1–628) acts as a DN mutant for HTLV-1 budding.

## Methods

### Cells

Human 293T cells were maintained in Dulbecco's minimal essential medium (Sigma, St. Louis, MO) supplemented with 10% fetal bovine serum and penicillin-streptomycin at 37°C.

### Plasmid construction

After PCR amplification from the K30 infectious clone, the HTLV-1 K30 gag gene was cloned into the pCAGGS-MCS vector. pVps4A, pVps4B, pVps4AEQ, and pVps4BEQ were described previously [12]. pCAGGS-HA-AIP1, -AIP1(1–628), and -AIP1(424–628) were kind gifts from Dr. Sakaguchi [30].

### VLP budding assay

Forty-eight hours after transfection, the cell supernatant was clarified from cell debris by centrifugation (13,000 × g, 10 min) and then VLPs were pelleted by ultracentrifugation through a 20% sucrose cushion (345,000 × g, 60 min at 4°C). Cells and VLPs were lysed with Lysis A buffer (1% TritonX-100, 25 mM Tris-HCl, pH 8.0, 50 mM NaCl, and 10% Na-deoxycholate). Cell lysates and VLPs were resolved by SDS-PAGE, and the proteins were then transferred onto nitrocellulose membranes. The mouse anti-HTLV-1 p19 monoclonal antibody TP-7 (Abcam, Cambridge, UK) was used to detect K30Gag. The mouse anti-Flag monoclonal antibody M2 (Sigma) was used for detection of Vps4A, Vps4B, Vps4AEQ, and Vps4BEQ. The mouse anti-HA monoclonal antibody 6E2 (Cell Signaling Technology, Beverley, MA) was used for detection of HA-AIP1 WT and DN series. Horseradish peroxidase-conjugated goat anti-mouse IgG antibody A-2304 (Sigma) was used as a secondary antibody. Immunoreactive bands were visualized using ECL plus (Amersham Pharmacia Biotech, Upsalla, Sweden), followed by the LAS-3000 system (Fuji Film, Tokyo, Japan). For quantification, the signal intensity on western blots was evaluated with Image Gauge version 4.1 (Fuji Film) using the LAS-3000 system.

## Competing interests

The author(s) declare that they have no competing interests.

## Authors' contributions

SU designed the study, carried out experiments, participated in analysis of the results, and wrote the manuscript. HY helped in drafting the manuscript and performed critical revisions. JY designed the study, participated in analysis of the results, and helped to draft the manuscript. All authors have read and approved the final manuscript.
